# Transverse and longitudinal right ventricular fractional parameters derived from four-chamber cine MRI are associated with right ventricular dysfunction etiology

**DOI:** 10.1038/s41598-023-32284-2

**Published:** 2023-03-30

**Authors:** Makito Sato, Tomoko Kato, Miyuki Ito, Yoko Watanabe, Junko Ito, Chisato Takamura, Masahiro Terashima

**Affiliations:** 1 Cardiovascular Imaging Clinic Iidabashi, Shin-Ogawamachi 1-14, Shinjuku-ku, Tokyo, 162-0814 Japan; 2 Department of Cardiology, International University of Health and Welfare Narita Hospital, Chiba, 286-8520 Japan

**Keywords:** Cardiology, Cardiovascular diseases

## Abstract

Studies of the usefulness of transverse right ventricular (RV) shortening are limited. We retrospectively analyzed the CMR images of 67 patients (age: 50.8 ± 19.0 years; men: 53.7%; Control: n = 20, Overloaded RV (atrial septal defect): n = 15, Constricted RV (pericarditis): n = 17, Degenerated RV (arrhythmogenic right ventricular cardiomyopathy): n = 15) (all enrolled consecutively for each disease) in a single center. We defined RV longitudinal (fractional longitudinal change: FLC) and transverse (fractional transverse change: FTC) contraction parameters. We assessed the FTC/FLC (T/L) ratio on four-chamber cine CMR views and compared the four groups regarding the fractional parameters. FTC had a stronger correlation (R^2^ = 0.650; *p* < 0.001) with RV ejection fraction than that with FLC (R^2^ = 0.211; *p* < 0.001) in the linear regression analysis. Both FLC and FTC were significantly lower in the Degenerated RV and Constricted RV groups compared with those in the Control and Overloaded RV groups. The T/L ratio was significantly lower in the Degenerated RV group (*p* = 0.008), while the Overloaded RV (*p* = 0.986) and Constricted RV (*p* = 0.582) groups had preserved T/L ratios, compared with the Control group. Transverse shortening contributes to RV function more significantly compared with longitudinal contraction. Impaired T/L ratios may reflect RV myocardial degeneration. RV fractional parameters may help precisely understand RV dysfunction.

## Introduction

Cardiovascular magnetic resonance (CMR) imaging is becoming an increasingly important tool in routine clinical practice, and this imaging is being applied for right ventricular (RV) volumetric assessment as the gold standard^1,2^. CMR enables visualization of a complete four-chamber view without deficits, while echocardiography sometimes fails to visualize the part of the RV free wall that is located near the rib cage. However, RV volumetric assessment by CMR requires certain equipment and intensive labor for segmentation^3,4^. Therefore, clinically, RV volumetric assessment by CMR is difficult to perform routinely.

**Figure 1:**
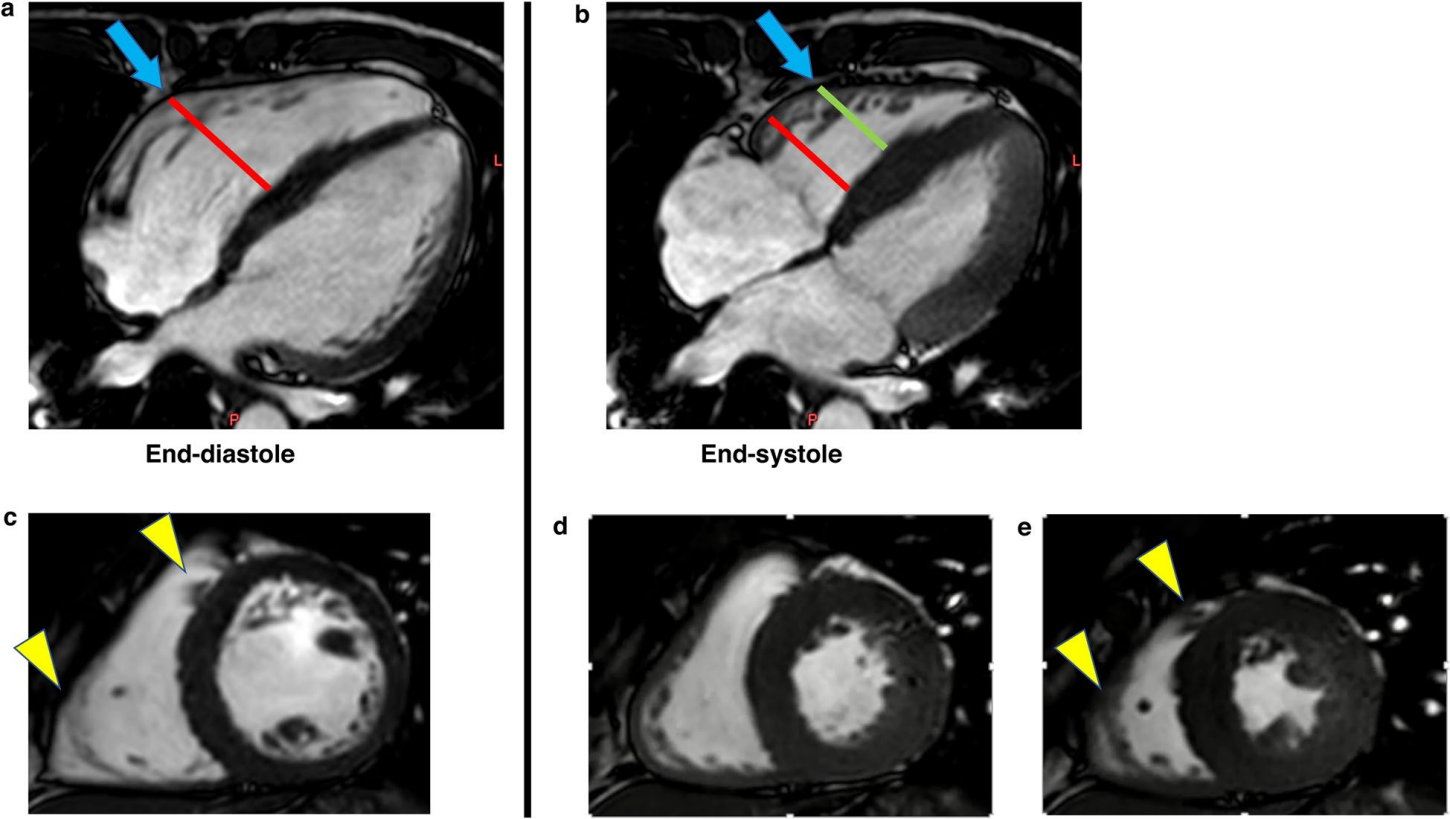
RV migration in the fixed LV short axis slice. Cine CMR of a subject with no structural abnormalities in the RV. In the four-chamber views, the RV portion marked by the blue arrows migrates in the cardiac cycle (**a**, **b**). (**c**) The LV short axis view along the red reference line in diastole. (**d**) The LV short axis view along the red reference line in systole. Circumferential strain was assessed between (**c**, **d**) on the same red reference line, regardless of the RV longitudinal shortening. (**e**) The LV short axis view along the green reference line in systole. Between (**c**) and (**e**), more significant circumferential shortening (than that seen in (**d**)) and the same structure of the RV (yellow visual cues) are visible. *RV* Right ventricle, *LV* Left ventricle, *CMR* Cardiac magnetic resonance.

In healthy subjects, the RV generates pressure mainly by longitudinal shortening^5^. In contrast, in subjects with structural abnormalities in the RV, etiology-specific shortening patterns (e.g., degenerated RV vs. overloaded RV) have been reported rarely. Currently, the evaluation of strain assessment on CMR using feature tracking is increasing in clinical practice^6^. RV longitudinal strain has prognostic importance in various cardiac diseases^7^. Furthermore, while the clinical application of RV circumferential strain appears to be limited compared with that of longitudinal strain, the usefulness of evaluating RV circumferential strain has been reported in patients with pectus excavatum^8^ and in some SLE cases^7^. Through-plane motion may affects 2D measures of circumferential strain using a fixed imaging plane (Fig. 1). It is also known in echocardiography that through plane motion affects LV circumferential strain in 2D measurements^9^. Strain analysis using 3D echo of the right ventricle has been utilized in recent years and has been reported to be effective as a prognostic factor^10^, but analysis also requires additional software and equipment and is not highly available at this time.

We preliminary defined parameters for evaluating the longitudinal and transverse shortening of the RV with high availability, and applied the parameters in subjects with various RV-related diseases. The aim of this study was to demonstrate whether these parameters correlate with RV ejection fraction and evaluate how parameters vary by RV-related disease.

## Methods

### Population

We retrospectively investigated the CMR data of patients with RV-related disease using clinical record databases. RV-related diseases were categorized into four groups: control, constricted RV (pericarditis), overloaded RV (atrial septal defect: ASD), and degenerated RV (arrhythmogenic right ventricular cardiomyopathy: ARVC). The data for consecutive subjects were collected for each etiology of RV structural abnormality. All subjects were recruited in a single center (the Cardiovascular Imaging Clinic Iidabashi) and all were recruited consecutively in each group over a specified period for that group (as noted in the following sections). Considering RV disease rarity, the target sample size was set at approximately 20.

The patients’ characteristics were obtained from the medical records, and all patients provided informed consent. All experiments were performed in accordance with the Declaration of Helsinki. The study was approved by the local ethical committee of the Cardiovascular Imaging Clinic Iidabashi Medical Corporation.

### Inclusion/exclusion criteria and disease diagnosis

Subjects referred by local medical institutes suspected as having any cardiac disease, but in whom cardiac disease was ruled out on CMR, with reported RV volumetric assessment, were recruited as the Control group. Subjects with constricted RV (pericarditis) were eligible if they had present or past symptoms of right heart failure, or were suspected of having pericarditis by computed tomography, and if marked thickening of the pericardium (maximum > 3 mm) was recognized on CMR. Subjects with overloaded RV (ASD) were eligible if left-to-right shunting was recognized in the interatrial septum, and the Qp/Qs ratio was > 1.5 by the phase-contrast method. Subjects with Ebstein anomaly were excluded. Subjects with degenerated RV (ARVC) were eligible if RV enlargement, asynergy, or fatty infiltration were recognized, they met at least one major or minor criteria of the 2010 task force criteria by CMR^11^ (subjects with a “Not probable” classification were excluded), and they were strongly suspected of having ARVC in the CMR assessment. In all groups, subjects with images of poor quality or inadequate four-chamber view cine CMR results were excluded from the study. Each diagnosis was made by cardiologists certified by the Japan Cardiovascular Society who had each read more than 1,000 CMR images.

### Patient enrollment

We enrolled 20 consecutive subjects as the Control group, in accordance with the inclusion criteria. CMR was performed from June 2018 to September 2018 for this group. There were no excluded cases owing to poor image quality. Twenty-three subjects with ASD were identified in the clinical records from June 2016 to Aug 2020. After excluding 8 subjects (Ebstein anomaly: 1, poor image quality owing to atrial fibrillation: 1, Qp/Qs < 1.5: 6), we enrolled 15 subjects in the Overloaded RV group.

Twenty consecutive subjects with chronic pericarditis were identified in the clinical records from June 2017 to February 2020. After excluding 3 subjects (pericardial thickness < 3 mm: 2, poor image quality: 1), we enrolled 17 subjects in the Constricted RV group. Adhesions of the pericardium on cine or tagged images were identified in 14 subjects. Symptoms (i.e., ascites, leg edema, shortness of breath) were seen in 14 subjects. Two subjects were suspected as having coronary artery disease, and another 3 subjects had histories of cardiac surgery (valvular disease: 2, coronary artery bypass graft: 1). Eighteen consecutive subjects suspected of having ARVC were identified in the clinical records from August 2016 to August 2020. After excluding 3 subjects (determined as “Not probable” in accordance with the task force criteria: 1, poor image quality: 2), we enrolled 15 subjects in the Degenerated RV group. Nine subjects were classified as definite, 4 subjects were classified as borderline, and 2 subjects were classified as probable in accordance with the 2010 task force criteria for ARVC. A total of 67 subjects were finally enrolled in the present study.

### Image acquisition

All examinations were performed using 1.5-T MRI scanners (Philips Achieva or Canon Vantage TITAN). The scan protocol was as follows: (Phillips/Canon): steady-state free precession cine sequence, 20/24 phase/beat, slice thickness: 8 mm, repetition time: 2.7/4.2 ms, echo time: 1.4/2.1 ms, flip angle: 60°/80°, field of view: 3 8 × 38/38 × 34 cm, and matrix: 208 × 193/192 × 240. The short axis slice was obtained with a 0 mm slice gap while the four-chamber view was generally obtained as a single slice, and other views (T2-weighted black blood, magnetic resonance coronary angiography, adenosine triphosphate-induced stress-perfusion imaging, late gadolinium enhancement) were performed in accordance with current guidelines^2^ and each patient’s status. Cine four-chamber view slices were obtained by referencing the left ventricular (LV) short axis and two-chamber views. After the determination of the axis between the center of the mitral valve and the apex with the two-chamber view, a four-chamber view slice that passed across the acute margin of the RV free wall was identified. Short-axis cine images were used for the RV volumetric assessment.

### LV and RV volumetric assessment

A workstation (Aquarius iNtuition, Ver. 4.4.13; Terarecon Inc.) was used for post-processing of the RV volumetric data. We calculated RV end-diastolic volume (RVEDV), end-systolic volume (RVESV), stroke volume (RVSV), and ejection fraction (RVEF). Papillary muscle volume was included as part of the blood pool. Another workstation, Zio2 Cardiac Function, was used for the LV volumetric assessment. We calculated LV end-diastolic volume (LVEDV), end-systolic volume (LVESV), ejection fraction (LVEF), stroke volume (LVSV), and LV myocardial mass. Volumetric data were indexed (I) by body surface area and labeled as RVEDVI, RVESVI, RVSVI, LVEDVI, LVESVI, LVSVI, and LV-myocardial mass index.

### RV fractional parameters

**Figure 2:**
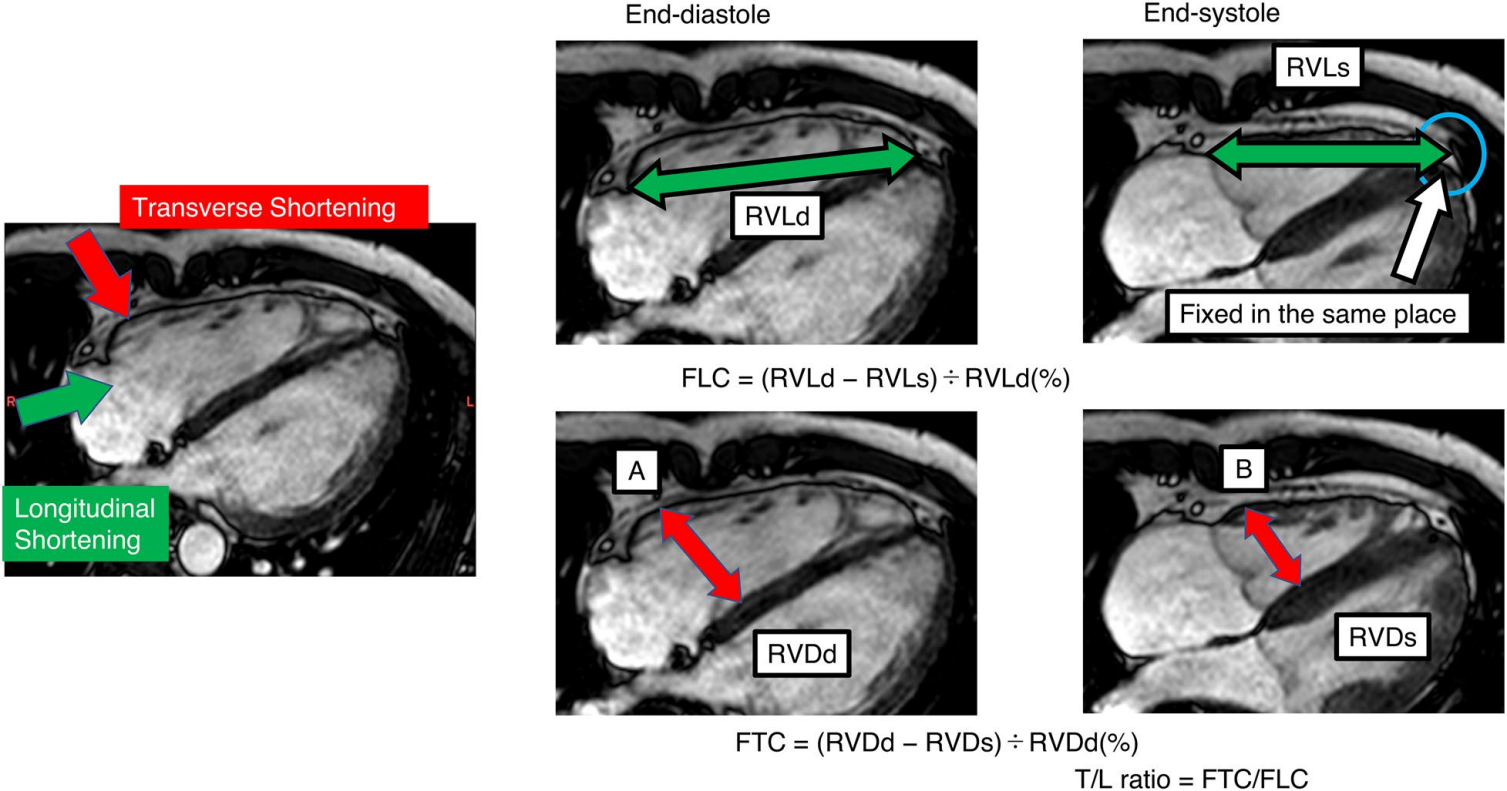
Measurement of fractional longitudinal change (FLC) and fractional transverse change (FTC). FLC = (RVLd − RVLs)/RVLd × 100(%), where RVLd is the RV length in end-diastole, and RVLs is the RV length in end-systole. RV length was defined as the distance from the RV apex to the attachment of the trigeminal valve in the RV free wall. We designated the location of the apex in end-systole as the same as that in end-diastole to address ostensible migration of the apex, which may cause overestimation of longitudinal contraction. We considered FLC to represent the parameter of longitudinal contraction. FTC = (RVDd − RVDs)/RVDd × 100(%), where RVDd is the RV diameter at end-diastole, and RVDs is the RV diameter at end-systole. The RV diameter was defined as the maximum distance between the septum and the RV wall, parallel to the annulus of the trigeminal valve. Points “A” and “B” were identified using four-chamber cine CMR and were recognized as almost the same portion of the RV in most cases. We considered FTC to represent the parameter of transverse contraction. The T/L ratio was determined to assess individual RV contraction patterns, as follows: T/L ratio = FTC/FLC. *T/L* Transverse/longitudinal, *CMR* Cardiac magnetic resonance imaging.

The RV shortening parameters were calculated in the cine four-chamber view retrospectively, and the parameters were defined as follows. Figure 2 shows the detailed methods for obtaining the measurements:

$$ {\text{Fractional}}\;{\text{longitudinal}}\;{\text{change}}\;\left( {{\text{FLC}}} \right) = \left( {{\text{RVLd}}{-}{\text{RVLs}}} \right)/{\text{RVLd}} \times {1}00\left( \% \right), $$

where RVLd is the RV length at end-diastole, and RVLs is the RV length at end-systole. RV Length was calculated as the distance from the RV apex to the attachment of the tricuspid valve in the RV free wall. We defined the location of the apex at end-systole as the same as that at end-diastole to address ostensible migration of the apex, which may cause overestimation of longitudinal contraction. We considered FLC to represent the parameter of longitudinal contraction

$$ {\text{Fractional}}\;{\text{transverse}}\;{\text{change}}\;\left( {{\text{FTC}}} \right) = \left( {{\text{RVDd}}{-}{\text{RVDs}}} \right)/{\text{RVDd}} \times {1}00\left( \% \right), $$

where RVDd represents the RV diameter at end-diastole, and RVDs represents the RV diameter at end-systole.

The RV diameter was defined as the maximum distance between the septum and the RV wall parallel to the annulus of the trigeminal valve. We considered FTC to represent the parameter of transverse shortening.

We calculated the FTC/FLC (transverse/longitudinal (T/L)) ratio to assess individual RV shortening patterns.

### Statistical analysis

First, we compared the fractional parameters and RV volumetric parameters among the four groups (i.e., Control, Constricted RV, Overloaded RV, Degenerated RV) to examine whether these parameters have the same tendency as RVEF among disease groups using Tukey’s multiple comparison procedure. Second, we compared the T/L ratio between the four groups using Tukey’s multiple comparison procedure to clarify the differences in contraction patterns between the groups. Third, we examined which parameter, FLC or FTC, showed the highest correlation using linear regression analysis for all subjects. All data were analyzed using Stata 15 (Stata Corp LLC). Continuous variables were expressed as mean ± standard deviation, and differences between the four groups were analyzed using Tukey’s test for multiple comparisons. Assuming that RV contractility comprises longitudinal and transverse shortening, the relationships between RVEF and FLC or FTC were evaluated using single and multiple linear regression analyses. Intra- and interobserver reproducibility of the measurements of FLC and FTC were assessed in 10 patients by calculating the intraclass correlation coefficients (ICC). *p* < 0.05 was defined as statistically significant.

## Results

### Patient characteristics

The patients’ characteristics are shown in Table 1. The subjects in the Constricted RV group were significantly older compared with those in the Control group; however, there was no statistically significant difference compared with the other groups. Regarding gender, the Constricted RV and Degenerated RV groups had higher male populations compared with the Control and Overloaded RV groups. Subjects in the Overloaded RV group had the highest body mass index among the groups.

**Table 1:** Patient characteristics, and volumetric and preliminary parameters of each of the four groups.

Characteristic/parameter	Control	Overloaded RV	Constricted RV	Degenerated RV	Summary of the between-group comparisons
	(n = 20)	(ASD, n = 15)	(Pericarditis, n = 17)	(ARVC, n = 15)	
Age (year)	44.0 ± 16.9	49.7 ± 20.0	62.6 ± 18.1	48.0 ± 17.5	Constricted > control; no other significant differences among the groups
Gender male, n, (%)	7 (35)	3 (20)	14 (82)	12 (80)	Degenerated and constricted > control and overloaded
BMI	21.4 ± 2.1	23.7 ± 2.8	20.8 ± 1.6	20.5 ± 2.2	Overloaded > control, constricted, and degenerated
Heart rate (bpm)	65.0 ± 12.5	70.0 ± 11.5	74.2 ± 11.2	67.4 ± 16.9	No significant difference among the groups
LVEDVI (ml/m^2^)	65.6 ± 14.1	61.7 ± 11.0	55.4 ± 15.6	75.9 ± 23.4	Degenerated > constricted; no other significant differences among the groups
LVESVI (ml/m^2^)	26.1 ± 7.8	21.7 ± 7.0	25.3 ± 12.2	35.6 ± 19.9	Degenerated > overloaded; no other significant difference among the groups
LVEF (%)	60.9 ± 5.6	63.8 ± 5.5	55.7 ± 9.0	54.6 ± 9.2	Overloaded > constricted and degenerated; no significant difference with control
LVSVI (ml/m^2^)	39.5 ± 7.8	39.3 ± 8.0	30.1 ± 6.7	40.1 ± 7.7	Control, overloaded, and degenerated > constricted
LVMMI (g/m^2^)	51.8 ± 8.3	43.2 ± 12.2	49.4 ± 11.2	53.2 ± 8.1	Degenerated > overloaded; no other significant differences among the groups
RVEDVI (ml/m^2^)	72.2 ± 14.3	114.6 ± 20.0	64.6 ± 15.1	125.9 ± 27.6	Degenerated and overloaded > control and constricted
RVESVI (ml/m^2^)	31.3 ± 7.8	49.9 ± 12.8	34.0 ± 11.1	83.9 ± 29.9	Degenerated > overloaded > control and constricted
RVEF (%)	56.7 ± 3.4	56.7 ± 6.6	48.7 ± 7.3	34.8 ± 10.4	Control and overloaded > constricted > degenerated(#)
RVSVI (ml/m^2^)	40.7 ± 7.4	64.6 ± 11.8	30.7 ± 6.4	41.8 ± 9.1	Overloaded > control and degenerated > constricted
FLC (%)	23.4 ± 4.6	19.5 ± 4.6	12.7 ± 4.5	14.9 ± 4.2	Control and overloaded > constricted and degenerated
FTC (%)	27.9 ± 5.4	24.4 ± 4.5	17.2 ± 7.2	9.36 ± 6.14	Control and overloaded > constricted > degenerated(#)
T/L ratio	1.25 ± 0.44	1.31 ± 0.35	1.47 ± 0.79	0.65 ± 0.43	Control, overloaded, and constricted > degenerated

Continuous variables are expressed as mean ± standard deviation. Nominal variables are expressed as number (%). Differences between two of the four groups were analyzed using Tukey’s test for multiple comparisons. # Of the two preliminary parameters, FTC showed the same tendency as that of RVEF.

*BMI* Body mass index, *LVEDVI* Left ventricular end-diastolic volume index, *LVESVI* Left ventricular end-systolic volume index, *LVEF* Left ventricular ejection fraction, *LVSVI* Left ventricular stroke volume index, *LVMMI* Left ventricular myocardial mass index, *RVEDVI* Right ventricular end-diastolic volume index, *RVESVI* Right ventricular end- systolic volume index, *RVEF* Right ventricular ejection fraction, *RVSVI* Right ventricular stroke volume index, *FLC* Fractional longitudinal change, *FTC* Fractional transverse change, *T/L ratio* FTC/FLC ratio.

Table 1 shows the results of the volumetric assessment and the FLC and FTC values of each group. A summary of the comparisons between the groups using Tukey’s test for multiple comparisons (detailed p-values, mean differences, and 95% confidence intervals (CI) between any two groups) are shown in Supplementary Table S1.

Regarding LV function, the Degenerative RV group had higher LVEDVI values than those in the Constricted RV group as well as higher LVESVI values than those in the Overloaded RV group. There were no significant differences between the other groups for LVEDVI and LVESVI. LVEF was lower in the Degenerative RV and Constricted RV groups compared with that in the Overloaded RV group; however, there was no significant difference compared with the Control group. The Constricted RV group had the lowest LVSVI among the four groups.

Regarding RV function, the Overloaded RV and Degenerative RV groups had higher RVEDVI values than those in the Control and Constricted RV groups. The Degenerative RV group had the largest RVESVI among the four groups. The Overloaded RV and Degenerated RV groups had larger RVESVI values compared with the Control and Constricted RV groups. The Control and Overloaded RV groups had higher RVEF values compared with the Constricted RV group, while the Degenerated RV groups had the lowest RVEF among the four groups. Among the four groups, the Overloaded RV group had the largest RVSVI value, while the Constricted RV group had the lowest RVSVI value.

Regarding FLC and FTC values, the Control and Overloaded RV groups had significantly higher FLC values compared with the Constricted RV and Degenerative RV groups. The Constricted RV group had lower FTC values than those in the Control and Overloaded RV groups. The Degenerated RV group had significantly lower FTC values than those in the Constricted RV group, so that Degenerated RV had the lowest FTC value among the four groups. Finally, FTC showed the same tendency as that of RVEF (Table 1).

**Figure 3:**
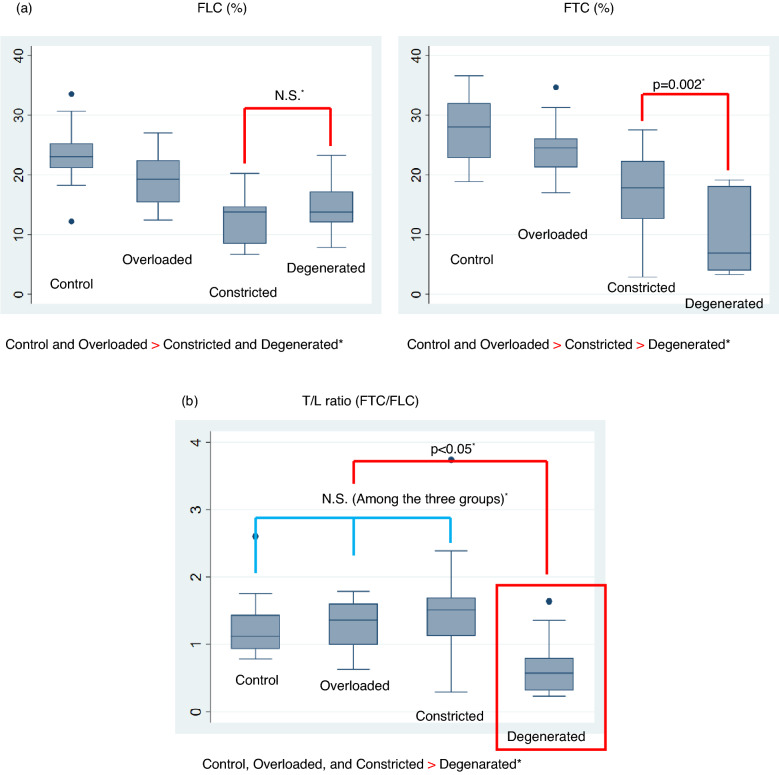
(**a**) Box plot of the FLC and FTC values in the four groups. The dots indicate outliers. Inequality signs indicate statistically significant differences between the four groups. The Control and Overloaded RV groups had higher FLC values than those of the Constricted RV and Degenerated RV groups. The Constricted RV group had lower FTC values than those of the Control and Overloaded RV groups. The Degenerated RV group had the lowest FTC value among the four groups. *Tukey’s test for multiple comparisons. (**b**) Box plot of the T/L ratios in the four groups. The dots indicate outliers. Inequality signs indicate statistically significant differences between the four groups. The Degenerative RV group had the lowest T/L ratio among the four groups, with an average T/L ratio of < 1 (FTC < FLC). *Tukey’s test for multiple comparisons. *FLC* Fractional longitudinal change, *FTC* Fractional transverse change, *T* Transverse, *L* Longitudinal, *RV* Right ventricle.

Regarding the T/L ratio, the Degenerated RV group had the lowest ratio among the four groups, with a T/L ratio of < 1 (FTC < FLC). Figure 3 shows a box plot for the FLC and FTC values, and the T/L ratios of each group.

**Figure 4:**
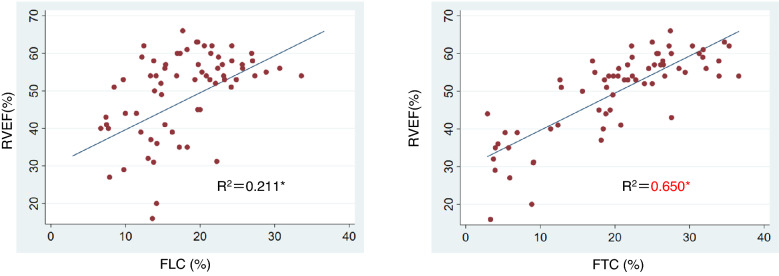
Scatter plots and linear regression lines for the preliminary parameters, FLC and FTC, versus RVEF. Univariate linear regression analysis revealed a mild correlation between FLC and RVEF (R^2^: 0.211; *p* < 0.001), while the correlation between FTC and RVEF was strong (R^2^: 0.650; *p* < 0.001). *Univariate linear regression analysis. The results of the multiple linear regression analysis are shown in Table 2. *RVEF* Right ventricular ejection fraction, *FLC* Fractional longitudinal change, *FTC* Fractional transverse change.

Figure 4 shows the scatter plots and regression lines between RVEF and FLC or FTC for all cases. Univariate linear regression analysis revealed a mild correlation between FLC and RVEF (R^2^: 0.211; *p* < 0.001), while the correlation between FTC and RVEF was strong (R^2^: 0.650; *p* < 0.001). As shown in Table 2, multiple regression analysis (dependent variable: RVEF, explanatory variables: FLC and FTC) implied that FTC (β coefficient: 0.937, 95% CI 0.730–1.144; *p* < 0.01) was a stronger explanatory variable compared with FLC (β coefficient: 0.139, 95% CI − 0.169 to 0.448; *p* = 0.37).

**Table 2:** Multiple regression analysis of FLC and FTC (dependent variable: RVEF).

	β-coefficient	95% CI	*p* value
FLC	0.139	− 0.169 to 0.448	0.37
FTC	0.937	0.730 to 1.144	< 0.001
Intercept	28.3	23.1 to 33.5	

*CI* Confidence interval, *RVEF* Right ventricular ejection fraction, *FLC* Fractional longitudinal change, *FTC* Fractional transverse change.

**Figure 5:**
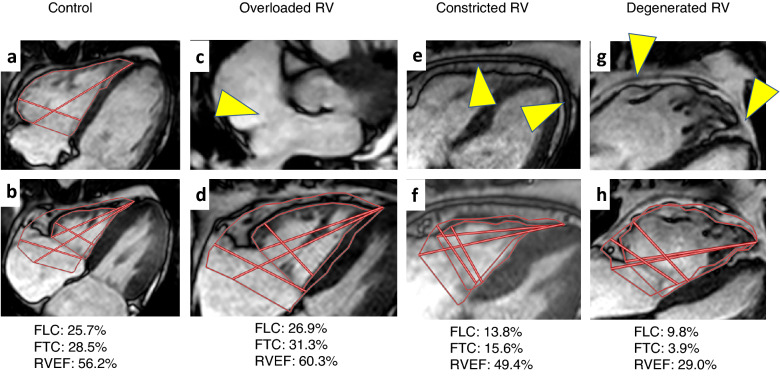
Representative cases. The straight red lines indicate the measured locations for RVLd, RVLs, RVDd, and RVDs. The polygonal lines indicate the endocardium of the RV at end-diastole and end-systole. (**a**, **b**) Four-chamber cine CMR of a woman in her 40 s; (**a**) end-diastole; (**b**) end-systole. CMR revealed no morphological abnormalities in the RV. (**c**, **d**) A woman in her 40 s with a history of ventricular tachycardia. CMR revealed an atrial septal defect and a left-to-right shunt (**c**, arrow). The Qp/Qs ratio was estimated at 2.2 by the phase-contrast method. (**e**, **f**) A man in his 70 s with right heart failure. Thickened pericardium is visible (**e**, arrow). (**g**, **h**) A man in his 40 s with a history of ventricular tachycardia. Remarkable impairment of RV contraction, and bulging and fat infiltration of the RV (**g**, arrow) are visible. *RVLd* Right ventricular length at end-diastole, *RVLs* Right ventricular length at end-systole, *RVDd* Right ventricular diameter at end-diastole, *RVDs* Right ventricular diameter at end-systole, *CMR* Cardiac magnetic resonance imaging.

Regarding the intraobserver reproducibility, the ICC was 0.903 (95% CI 0.677–0.974) for FLC and 0.961 (95% CI 0.861–0.990) for FTC. The time required to measure these parameters was 67.1 ± 15 s for operator A. Regarding interobserver reproducibility, the ICC was 0.819 (95% CI 0.453–0.951) for FLC and 0.894 (95% CI 0.653–0.972) for FTC. The time required to measure these parameters was 168 ± 28 s for operator B. Representative cases are shown in Fig. 5.

## Discussion

Our study demonstrated that the RV parameters, FLC and FTC, can evaluate RV shortening from a new perspective. Fractional shortening parameters for RV have been studied using echocardiography, and all studies showed moderate correlation with MRI-derived RV function^12,13^. MRI can visualize the entire morphology of the RV by cine stack; therefore, there may be controversy regarding the necessity of RV fractional parameters derived using only single-slice images. However, RV functional parameters derived by two-dimensional echocardiography were reported to be poorly correlated with MRI-derived RV volume, and these correlations improved only modestly when two-dimensional measurements were performed using MRI^14^. High intra- and interobserver reproducibility of LV and RV volume measurements on CMR was reported in several studies. However, these measurements require more than 20–30 min per case^3,4^. Therefore, some institutes are unable to analyze RV volumetry in all cases in clinical practice. In comparison, measurements of the fractional parameters described in the present study can be obtained within 2 min by a skilled operator, as shown in our results, without additional equipment. Left ventricular stroke volume should be used to assess the validity of RV volume analysis ^2^, which is difficult in the presence of valvular disease or intracardiac shunts. This new parameter may be able to predict right ventricular contractility within a certain range in every situation, and correct for errors in the analysis by measuring it beforehand.

**Figure 6:**
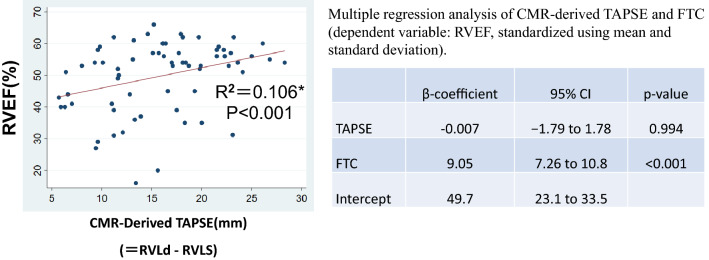
Scatter plots and linear regression lines for CMR-derived TAPSE, versus RVEF. CMR-derived TAPSE was defined as RVLd-RVLs. *Univariate linear regression analysis. The results of the multiple linear regression analysis are shown in the right side. In univariate regression analysis, CMR-derived TAPSE showed a weak correlation to RVEF. And it failed to show a significant correlation in a multiple regression analysis with the FTC. *TAPSE* Tricuspid annular plane systolic excursion, *RVLd* Right ventricular length at end-diastole, *RVLs* Right ventricular length at end-systole, *CMR* Cardiac magnetic resonance imaging.

TAPSE (tricuspid annular plane systolic excursion) is a very widely used measure of RV longitudinal contraction as a standard. There have been some reports evaluating right ventricular function using CMR-derived TAPSE^15^. We defined “CMR-derived TAPSE” as “RVLd-RVLs”, and ran the same analysis (scatter plot and multivariable regression analysis), replacing FLC with CMR-derived TAPSE (Fig. 6). In univariate regression analysis, TAPSE showed a weaker correlation to RVEF (R^2^ = 0.106, *p* < 0.001) than FLC (R^2^ = 0.211, *p* < 0.001). The target population of the present study has a large variation in RV size. In such cases, standardizing the excursion distance by the length of the entire RV may lead to a more accurate assessment of contractility. And CMR-derived TAPSE failed to show a significant correlation in a multiple regression analysis with the FTC, as well as FLC.

The present study also demonstrated that both longitudinal and transverse shortening play important roles in RV contraction, especially in subjects with RV-related disease. The RV myocardial structure mainly comprises two layers^16^. On the epicardial surface, circumferentially-oriented fibers are present, which are shared with LV epicardial myofibers. In contrast, subendocardial myofibers form longitudinal lines, and these fibers are mainly responsible for longitudinal shortening, which generates the largest part of RV contraction in healthy subjects^17^. However, once RV pressure overload occurs, it may lead to ventricular hypertrophy and morphological changes to reduce the wall stress. Under these circumstances, the dominance of the circumferential fibers can be observed^16^. A comparative study of echocardiography and CMR involving subjects with precapillary pulmonary hypertension showed that correlations between tricuspid annular plane systolic excursion or fractional longitudinal wall motion and RVEF were relatively mild when compared with correlations between functional area change and RVEF^18^. These findings indicate that both longitudinal contraction and transverse contraction must be considered in subjects with RV dysfunction or pulmonary hypertension.

As mentioned, our preliminary parameter, FTC, can determine maximum RV diameter visually in each cardiac phase on four-chamber cine CMR, and using the FTC value, we can minimize the influence of longitudinal shortening. Using this method, rating the fractional shortening parameters may pioneer disease- or individual-specific RV function analysis beyond conventional volumetry. Additionally, FLC may be approximated to RV longitudinal strain measured by feature tracking. A meta-analysis by Vo et al. showed that RV longitudinal strain varied between studies, and the mean value derived using a random effects model was − 21.8%^6^, similar to the FLC value of the Control group in the present study of 23.4% ± 4.6%.

In our present study, significant transverse shortening was seen in control subjects (mean FLC: 23.4%, mean FTC: 27.9%; Table 1). The result has discrepancy with the previous reports^5^. We agree that the largest component of RV contraction in healthy subjects is longitudinal contraction. One of the ideas to resolve this discrepancy is that longitudinal contraction is not perpendicular to the AV valve, so the force is distributed vertically and parallel (circumferential) to the annular surface. This creates transverse shortening even in normal subjects. In addition, Circumferential (or transverse) muscle tension will be essential as a supplementary factor even in healthy subjects with longitudinal-dominant contraction (supplementary Fig. S1).

Constrictive pericarditis (CP) is a condition in which the loss of pericardial elasticity causes restriction on diastolic filling of the heart^19^. Currently, the diagnosis of CP is made on the basis of clinical features and echocardiography. Cardiac MRI is recommended in patients for whom echocardiography is non-diagnostic. A threshold of pericardial thickness of > 3–4 mm on CMR yielded a sensitivity and specificity of 83%–91% and 100%, respectively, to diagnose CP, in one study^20^. Some reports described impairment of RV systolic dysfunction in subjects with CP, and stated that this finding may indicate persistent RV dysfunction after pericardiectomy^19,20^. In these reports, RV atrophy caused by immobilization owing to prolonged constriction was considered a mechanism of RV dysfunction. In our CMR study, a high prevalence of adhesions between the myocardium and pericardium was seen in the Constrictive RV group, which had lower LVSVI and RVSVI values than those in the other groups. Additionally, the Constrictive RV group demonstrated remarkable impairment of FLC and FTC compared with the Control group. Immobilization or adhesion of part of the myocardium may cause RV dysfunction, expressed by FTC or FLC, and impairment of bi-ventricular stroke volume.

ASD is the most common congenital heart disease with left-to-right shunting in adults^21^. Defects with shunts with Qp/Qs ratios > 1.5 are considered significant and are responsible for different degrees of right heart volume overload^22^. Our study demonstrated that the Overloaded RV group had preserved RVEF and the same range of contraction parameter (FLC/FTC) values as those of the Control group, and there were statistically significant differences in T/L ratios in the Overloaded RV group compared with those in the Degenerative RV group. These findings indicate that FLC, FTC, and T/L ratio can distinguish Overloaded RV and Degenerated RV, both of which present with RV enlargement.

ARVC, which we classified as Degenerated RV, is a genetically determined heart muscle disorder that is characterized pathologically by fibro-fatty replacement of the right ventricular myocardium. The distribution of this structural abnormality is described as the “triangle of dysplasia” (inflow tract, outflow tract, and apex of the RV)^11^. Te Riele et al. reported that “mutation-positive” ARVC describes a previously unrecognized characteristic pattern of disease involving the basal inferior and anterior RV, and the posterolateral LV, while the RV apex is involved in only advanced ARVC^23^. Our study, showing predominantly impairment of transverse contraction (FTC) compared with longitudinal contraction (FLC), may indicate some relationships with a specific distribution of lesions in subjects with ARVC.

**Figure 7:**
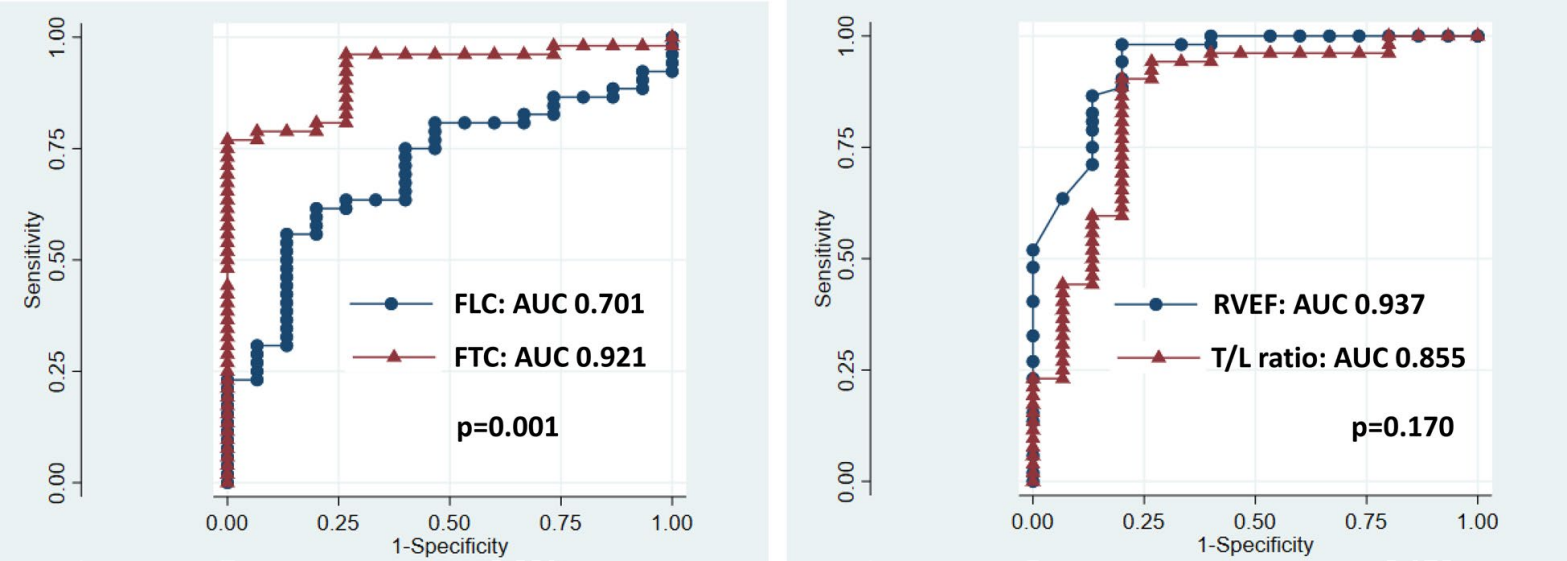
ROC curve for “Degenerated RV” detection. The left side: ROC curve for FLC and FTC. The right side: ROC curve for T/L ratio and RVEF. Figures indicate AUC for the parameters. *p* value indicate the probability of the null hypothesis in testing the equivalence of ROC curves. *ROC* Receiver operating characteristic, *AUC* Area under the curve, *RVEF* Right ventricular ejection fraction, *FLC* Fractional longitudinal change, *FTC* Fractional transverse change.

After showing the characteristics of FLC, FTC, and T/L ratio in the subjects with ARVC, we additionally examined how accurately the FLC, FTC and T/L can predict "Degenerative RV" using ROC curves for the entire population of present study (Fig. 7). Equality of ROC curves between FTC and FLC and between T/L ratio and RVEF was assessed based on the method proposed by Delong et al.^24^. AUC was 0.701 for FLC, 0.921 for FTC, 0.855 for T/L ratio, and 0.938 for RVEF, respectively. FTC was able to predict ARVC more accurately than FLC (*p* = 0.001), and T/L ratio showed comparable accuracy to RVEF (*p* = 0.170), considering that RVEF itself is included in the diagnostic criteria for ARVC, FTC and T/L ratio have high diagnostic power for “Degenerated RV”.

Our study has some limitations. First, this was a single-center, cross-sectional study. Additionally, the rarity of each disease resulted in small sample sizes. Second, as mentioned, we could not assess echocardiography simultaneously because our institution is an outpatient imaging center. Third, RV contraction is affected by pressure overload; therefore, relationships between RV contraction patterns (T/L ratio) and RV pressure overload should be investigated further. Furthermore, we couldn’t obtain the data of strain analysis due to the lack of technical equipment. Further comparative study of strain analysis and present parameters is needed for the assessment of accuracy of the present preliminary parameters.

In conclusion, transverse shortening plays an important role in RV contraction; more so than longitudinal shortening. Bi-directional RV fractional parameters are easily measured, reflect RV contraction to some extent, have the potential to differentiate the etiology of RV dysfunction and detect abnormal RV morphology.

## Supplementary Information

Supplementary Figure S1.

Supplementary Legends.

Supplementary Table S1.

## Data Availability

The data that support the findings of this study are available from the corresponding author (MS), upon reasonable request.
