# Supplementary figures and images for: Transverse and longitudinal right ventricular fractional parameters derived from four-chamber cine MRI are associated with right ventricular dysfunction etiology
Source: Sci Rep. 2023 Mar 30;13:5229. doi: 10.1038/s41598-023-32284-2 (PMC10063639; doi:10.1038/s41598-023-32284-2)

## Slide 1
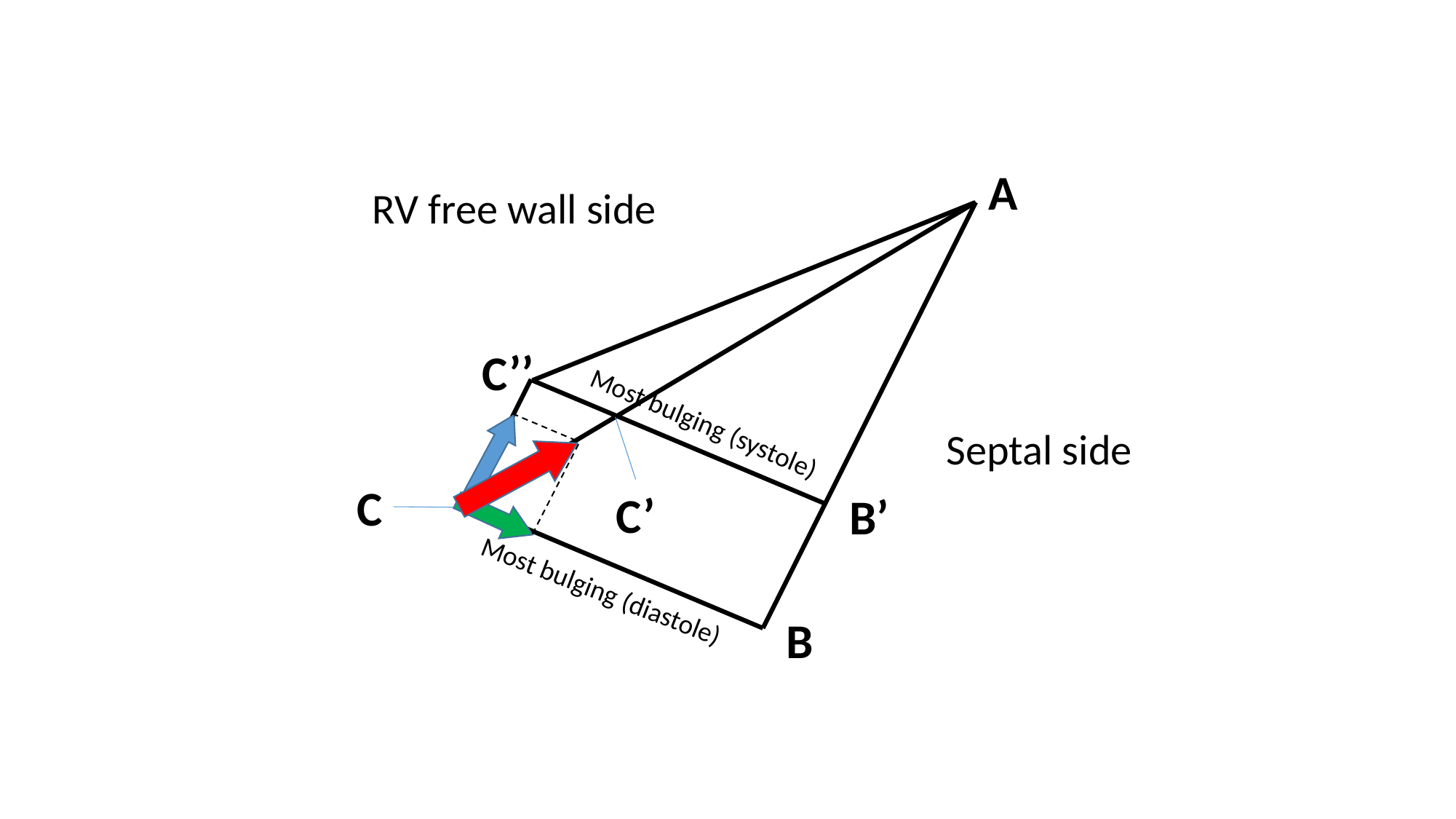

A
RV free wall side
C’’
Most bulging (systole)
Septal side
C
C’
B’
Most bulging (diastole)
B

Supplement: Supplementary file 1 — Supplementary Figure S1. [file 41598_2023_32284_MOESM1_ESM.pptx]
