# Supplementary material for: Transverse and longitudinal right ventricular fractional parameters derived from four-chamber cine MRI are associated with right ventricular dysfunction etiology
Source: Sci Rep. 2023 Mar 30;13:5229. doi: 10.1038/s41598-023-32284-2 (PMC10063639; doi:10.1038/s41598-023-32284-2)
Supplement: Supplementary file 2 — Supplementary Legends. [file 41598_2023_32284_MOESM2_ESM.docx]

**Figure S1.**

Schematic diagram of the right ventricle (4-chamber view) simplified into a triangle (diastole: ABC, systole: AB’C’). Longitudinal contraction occurs parallel to the wall surface (red arrow) and can be divided into forces parallel (blue arrow) and horizontal (green arrow) to the annulus surface. Horizontal forces may produce some of the transverse shortening.　If the circumferential muscle tension do not function(systole:AB’C’’), the width of most bulging RV part will not change through cardiac phase(BC→B’C’’), and RV free wall should appear to protrude outward during systole in 4 chamber view(AC→AC’’). But empirically, we seldom see such(systole:AB’C’’) RV in healthy subjects.
